# Periscope

**Published:** 1889-03

**Authors:** 


### PERISCOPE.

### MEDICINE.

**Weil's Disease.**—At a recent meeting of the Society for Internal Medicine, in Berlin, Professor A. Fraenkel brought forward a case of this disease. A medical student received, in a duel, a cut over the right frontal protuberance. Thirty-six hours after, when the wound had apparently healed by first intention, rigors set in, and the temperature ran up to 104° F. About forty-eight hours later, a redness about the size of a shilling appeared in the neighbourhood of the wound, disappearing after two days. The fever continued, and patient became restless, sleepless, and delirious; then jaundice appeared, with coated tongue, diarrhœa, with biliary pigment and albumen in the urine. Nine days later, a gradual rise of temperature to 103° F. occurred, with a corresponding gradual fall, while the convalescence took several weeks. This disease was first described by Weil two years ago, in four cases. Its chief symptoms, which are well seen in Fraenkel's case, were given thus:—Strong, well-built men are suddenly seized with rapidly increasing fever, great weakness, unquiet sleep, with tendency to somnolence and delirium; moderate jaundice soon occurs, with enlargement of the liver and spleen; albuminuria, coated tongue, and diarrhœa. This condition, with temperature to 105.5° F., lasts 6—8 days, the temperature then falling slowly to the normal. After a pause of 1—7 days, the temperature rises gradually, and by degrees falls again. Great prostration remains, and the convalescence lasts several weeks. When jaundice is present, bile colouring matter and biliary acids, blood-corpuscles and casts appear in the urine, and the motions are colourless. Weil considers it to be an absorption jaundice. The differential diagnosis lies between it and acute yellow atrophy of the liver, relapsing fever and some cases of typhoid. The state of the liver and the absence of a spirillum in the blood distinguishes it from the first two respectively; but in some cases of bilious typhoid the diagnosis is occasionally difficult. Weil looks upon the disease as a general affection caused by infection or intoxication, with consecutive swelling of the abdominal organs and their cellular elements. The origin of the virus is probably in the intestine. Fraenkel holds it is a form of septic infection, the place of inoculation differing in different cases. A. Baginsky related a case in a child thirteen months old; and Bartels agreed with Fraenkel in viewing the disease as of septic origin.—*Berliner klinische Wochenschrift*, No. 4, 1889.

**Rumination, or Merycismus.**—Researches into the etiology of this rare disease—only a hundred cases of which have been observed since Fabricius first described it, nearly three centuries ago—have been directed more particularly, of late years, to the examination of the stomach-secretions, in the hope that a chemical basis might explain the peculiar train of symptoms and furnish some indications for a rational treatment of the disorder. So far, however, the only constituent of the gastric fluid found to vary is the free hydrochloric acid; but to what extent this is to be looked upon as an etiological factor may be gathered from the following cases, recently published:

Dr. Alt gives a case from the "Nerven Klinik" of Professor Hitzig, of Halle (*Berliner klinische Wochenschrift*, Nos. 26 and 27, 1888). The patient was a man, 24 years old, who had suffered from his 10th year from regurgitation of food. At seven years of age he suffered from severe headaches, which affected his memory; and two years later, from chronic stomach-catarrh. His chief symptom was regurgitation of food or drink after a longer or shorter time, varying from a few minutes to many hours, or even days, after swallowing; it was then chewed thoroughly and re-swallowed. The food was usually bolted the first time: if he chewed it to any extent, it returned again, in a pulpy state, and tasted badly. His hunger was not appeased until after the second mastication; and if the interval between deglutition and regurgitation was at all long, he suffered from a feeling of pressure over the stomach and pyrosis. He attributed the disorder to swallowing large quantities of fruit untouched: thus, cherries swallowed whole were, after regurgitation, masticated and the stones spat out. Return of the food could not be readily induced; but sometimes, by inspiring very deeply and holding the breath, it succeeded. The patient appeared poorly nourished and had a furred tongue. There was a moderate degree of dilatation of the stomach, all the other organs being normal. A test-meal, consisting of soup, beef-steak, potatoes, bread, and some water, he was able to repress for five hours, during which he had increasing hunger and burning pain in the stomach. A tube was introduced with ease, and the stomach-contents removed and examined. These formed a thickish food-pulp, reacting strongly acid, giving the HCl action markedly, but containing no lactic acid; whilst it showed effective digestion of proteids and very little alteration in starchy food. Hyperacidity was present. To test some of the patient's statements and prove the deficient closure of the cardiac end of the stomach, the patient, after the stomach had been washed out, swallowed quarter of a litre of water containing two living gold-fish of 6½ and 5½ cm. in length: the fish could be heard splashing about in the stomach by applying the ear to the abdominal wall; twenty minutes afterwards, the larger gold-fish was returned into the mouth without any fluid, and fell into a vessel of water, in which it swam about very actively; eight minutes later, the smaller one was brought up in a similar way, apparently none the worse. Alt looks upon the hyperacidity as the essential factor. He says that in all the recorded cases the patients bolted their food; its admixture with the saliva being slight, the alkalinity was soon neutralised; a great quantity of acid being secreted, hyperacidity resulted; this stimulated peristalsis of the stomach-walls, and the cardiac end, being deficient, allowed the food to return into the mouth, where it was thoroughly mixed with the alkaline saliva, which neutralised the hyperacidity and allowed digestion of starch to go on. Treatment was adopted according to this view. There was a systematic emptying of the stomach of the undigested starch: a meal was given with proteids preponderating; while alkalies, especially carbonate of soda, were administered. In addition, the lower end of the œsophagus was galvanised by the introduction of a sound. This treatment gave great relief in 14 days; and two months after, patient was free from his former trouble.

A different alteration obtained in the stomach-contents in a case reported by Boas (*Berliner klinische Wochenschrift*, No. 31, 1888.) In this case, a man, ætat. 29, a slight but typical rumination developed a short time after an attack of acute gastritis inhis 18th year. Under the influence of dietetic errors and irregular habits, it became more pronounced and occurred oftener, gradually producing headaches, feeling of fatigue, incapacity for continuous work, depression, and latterly emaciation; objectively there was a high degree of hyperæsthesia of the abdominal walls, loss of patellar reflexes, marked sensibility to pressure over epigastrium; the stomach was of normal size, and the cardiac end was capable of closure. Regurgitation took place usually about a quarter of an hour to one hour after deglutition. The regurgitated food always tasted agreeably sweet, the patient ruminating openly with great pleasure. Chemical examination, carried out for three weeks, showed considerable decrease of free HCl. Twenty drops of pure HCl were given during every meal, and led to some improvement in the symptoms.

Still otherwise was the condition of the stomach-contents in two cases recently communicated by Jürgensen of Copenhagen. In the first case—a man 23 years old, no heredity and no nervous history—the affection began, two and a half years previously, by hiccough; then pieces of meat came up, followed soon by a development of the rumination during every meal. He had neither nausea nor dyspeptic symptoms. Repeated examinations after test-meals showed an entire absence of free HCl. Daily washing out of the stomach and the artificial introduction of food had but little influence on the course of the disease. His second case was a girl, aged 20, whose father and grandfather ruminated. When 10 years old, she began suddenly to ruminate. Regurgitation came on immediately at the close of a meal—sometimes even during its course, if a pause was made. There was no nausea, but a peculiar giddy feeling. All kinds of food rose up: milk, however, was best retained; while eggs, coffee, and fats were but a short time held down. There was an absence of free HCl in stomach-contents. The introduction of food by a tube into the stomach gave some slight relief, but the rumination soon set in again.

In all those cases the stomach-contents were of different chemical conditions—hyperacidity, subacidity, and want of acidity occurring in individual cases. From these facts, Boas believes that the affection is probably a stomach neurosis, and concludes that the chemical state of the mucous membrane of the stomach is not at all an essential factor, but merely an accidental occurrence in the complex of symptoms.—*Berliner klinische Wochenschrift*, Nos. 26, 27, 31, and 46, 1888.

D. R. PATERSON, M.D.

### THERAPEUTICS.

**Myrtol.**—This substance, which is a clear liquid of pleasant odour, is strongly recommended by Professor Eichhorst, of Zurich, in diseases of the respiratory tract, as a powerful disinfectant and deodorant. It is best administered in the form of capsules. In putrid bronchitis and gangrene of lung, a deodorising and disinfectant influence is obtained by the administration of 2—3 capsules every two hours; care being taken when three are given, owing to the tendency of loss of appetite to be produced. The action is, not infrequently, very rapid, a few capsules causing the disappearance of the horrible smell from the breath and expectoration. The quantity of expectoration decreases very rapidly; while in several cases which Eichhorst saw, cure resulted. Myrtol has no action on the tubercle-bacillus; for in a case of gangrene of lung, in which recovery took place, the bacilli appeared in the sputum notwithstanding the continued use of the drug. —*Therapeutische Monatshefte*, Heft i., 1889.

**Iodol in Internal Diseases.**—Cervesato of Padua, after an extended trial of this drug in various diseases, gives the following results in cases where he has found it useful:—

I. **In Scrofula.**—Its action is best seen in the torpid form, especially where the glandular swellings have not yet suppurated; to a lesser degree it was useful in strumous appearances of mucous membranes, especially of the nose and throat: it was least active in scrofulous dermatitis, as impetigo and eczema. Iodol was used in daily doses of 7—20 grains, according to the age of the child, for a period of 2—3 months. This was supplemented, in chronic adenitis, by the inunction of iodol ointment (one part iodol to fifteen parts vaselin); or insufflation of the powder, in cases of catarrh. It was well borne, causing no digestive disturbances.

II. **Diseases of Respiratory Organs.**—It is here given in 15—45 grains, and also by inhalation and insufflation. In advanced cases of lung tuberculosis with extensive cavities, there was no influence seen; but where there was primary laryngeal tubercle, a distinct improvement was noticed. It was useful in acute and subacute laryngitis, as insufflation. In a case of extensive catarrh of the smaller bronchioles, with recurring asthmatic attacks, internal use, together with inhalation, gave great relief. In two children with dry bronchitis, there was a profuse secretion following its action, affording great benefit; while in three cases of stationary pleural effusion in children, rapid absorption followed administration of the drug.

III. **Tertiary Syphilis.**—In two cases of gummata of the throat, the internal use of 30—45 grains daily, as well as local application, led to cure in two months. The local solution used was: iodol, 1; alcohol, 16; glycerine, 34. A case of tertiary syphilis, which affected the larynx and the liver, rapidly yielded to it.

Iodol is well borne; it has no influence on the body temperature, on the circulation or respiration, and it does not produce iodism.—*Berliner klinische Wochenschrift*, No. 2, 1889.

**Sozoiodol.**—Nitschmann confirms the favourable results obtained by the use of the soda preparation of this salt by Fritsche in affections of the naso-pharynx; instead, however, of insufflation, he has used it in the form of a solution, 5—7 per cent., applied with a brush. Langgaard has already shown its powerful action on germ-formation; while at the same time it is entirely free from any poisonous effect, which makes it useful in large abrasions of the surface. Nitschmann found it particularly useful in wounds where there is a good deal of suppuration; as in injuries from machinery, sloughing from molten metal, superficial burning, chronic ulcers of the leg, etc. It was much superior to iodoform in cases where the granulations were exuberant, and where there was free suppuration. The ointment of the soda preparation rapidly cleanses the wound, and keeps down the granulations. Comparing it with iodoform, in two cases of superficial burn of almost the same extent, he found that the one treated with sozoiodol healed twice as fast. It is used in the following form:

Sozoiodol Natr. ... ... 4 pts.
Lanolin ... ... ... ... 40 pts. Ft. ung.

In acute pharyngitis, as well as in acute stomatitis, a watery five per cent. solution of the zinc preparation, brushed on every two hours, acts quickly. In conjunctivitis purulenta and blenorrhœa neonatorum, a lotion like the following acted very well:

Sozoiodol Natr. ... ... ... ... 2 pts.

Aquæ ... ... ... ... ... ... 30 pts.

Zinc preparations cause burning pain. In gonorrhœa it is useful as a two per cent. solution, and in vaginitis it may be applied as an ointment on a tampon; but the disease, *par excellence*, for which it is to be recommended is catarrh of the cervix. Here it is used in the form of a powder. The cervix is laid bare with a speculum, and the powder is blown in through a glass tube, with indiarubber tubing attached to it. A dry tampon is then laid into it, to prevent the rapid solution of the substance. In cases of endometritis, a seven per cent. solution was injected into the uterus by means of a Braun's syringe, care being taken to withdraw the greater part of it after a short time, in order to avoid the fluid entering the tubes. Some improvement followed this. —*Therapeutische Monatshefte*, Heft. i., 1889.

**Toxic Effects of Antipyrin.**—Müller (*Schweizer Correspondenzblatt*, No. xviii., 1888) observes that after the use of this drug, not only does the expected action sometimes fail, but symptoms appear which are to be regarded as due to some influence on the nervous system : thus, exanthems of different forms and bleeding from the mucous tracts appear, complicated often by grave head-symptoms—coma and convulsions—and also attacks of heart-failure. On the other hand, a contrary effect is sometimes observed: neuralgic pains are increased, and become unbearable, while a slightly febrile temperature may rise some degrees after its use. In these cases, the continued administration may induce serious symptoms: thus, Müller had a case of joint-rheumatism in a ten-year-old child, where a single dose of 3—4 grains of antipyrin raised the temperature, and two or three more doses brought on brain-symptoms, heart-weakness, &c. These cases may be due to some idiosyncrasy.

Rapin of Geneva (*Revue Médicale de la Suisse Romande*, No. 11, 1888) reports a case where alarming symptoms occurred after a dose of 15 grains. The patient, aged 28, had taken the same dose several times before, for sciatica, and as relief followed, it was discontinued for five days. Scarcely had she taken it again, when violent burning pains in the stomach, vomiting, and collapse set in. The lips were cyanotic, the pulse small and rapid, so that the supervention of death was feared. Some hours later, an itching rash appeared over the whole body. Twenty-four hours afterwards patient was quite well. In two other patients (arterio-sclerosis, with angina), Rapin saw death occur after the use of 15 grains of antipyrin.—*Therapeutische Monatshefte*, Heft i., 1889.

**On the Therapeutic Effects of Washing out the Stomach in Infancy.**—Leo gave his experience before the Berlin Medical Society in this mode of treatment—first proposed by Epstein—in 104 cases. He used pure water alone, or with the addition of a few drops of a 20 per cent. thymol solution; and the effect, in all cases beginning with vomiting, was most striking. The vomiting disappeared completely, or was greatly lessened. In acute dyspepsia, with or without vomiting, a single washing-out often sufficed to cure it. The result in cholera infantum was not so marked; but in some cases it was of use, especially when combined with calomel and opium. In other cases of chronic and subacute stomach-catarrh, as well as in simple and sometimes even in extreme cases of diarrhœa, good effects were seen. A. Baginsky agreed with Leo in the good results to be got from washing out the stomach. He considered it contra-indicated in cases where there is grave inflammatory irritation of the bowels with high fever, great pain, and danger of peritoneal complication, as well as in cholera infantum after collapse has set in. It is, however, a sovereign remedy in habitual vomiting after ablactation—the most intractable cases yielding to it. In atonic conditions of the stomach and intestinal tract in rachitic children, this treatment is of great use. Simple water is quite effective; but in washing out the intestine, NaCl solution should be used, as the mucous membrane is very sensitive. Monti has shown that washing-out of the intestine is effective in affections high up in the canal, as seen in its effect on catarrhal jaundice. Professor Henoch endorsed this treatment. In most cases he obtained decided results; the introduction was easy, and he never saw any danger arise from it. Klein used the treatment in 30 cases of infantile cholera, making use of an ordinary funnel and catheter, with good success.—*Berliner klinische Wochenschrift*, Nos. 48 and 49, 1888. D. R. PATERSON, M.D.

**Phenacetin.**—In experiments on animals, phenacetin has been found, in doses of one gramme per kilogramme of the body-weight, to lower the temperature 1° C. in eight hours; double this dose has been given without producing toxic effects. Given in doses of 15—30 grains to a healthy man, it produces no effect on the temperature; given to a fever patient, it lowers the temperature in about 30 minutes. Its maximum effect is produced one hour or one and a-half hours after ingestion, and the mean duration of its action is four hours. It is said to produce no bad effects—neither cyanosis, like antifebrin, nor cutaneous eruptions, like antipyrin. Vertigo and shivering sometimes occur when the dose exceeds gr. xxx.—xlv. in twenty-four hours (Dujardin-Beaumetz). It has no effect on the pulse and respiration, and it is eliminated by the urine, the quantity of which is a little diminished during its administration. It has proved itself an efficient antipyretic in malarial fevers, pneumonia, acute rheumatism, and enteric fever; it is an excellent analgesic in neuralgia, and in painful affections in hysterical patients. It has been used with success in polyuria of nervous origin, but has no influence on diabetes. Dose as antipyretic gr. v. every four hours to commence with. Dose as analgesic, gr. viii.—x. It is best given in powder or capsule, or, in the case of children, mixed with the food. It is almost tasteless; is very insoluble, but dissolves in alcohol, ether, acetic acid, and sparingly in lactic acid at 30° C. This last reaction probably explains its solubility in the stomach.—*Le Progrès médical*, December 29th, 1888.

[I have used phenacetin in a large number of cases: it is an excellent antipyretic, rarely giving rise to toxic effects. The above statements, that it never gives rise to cyanosis or affects the pulse, are too absolute. In two cases of enteric fever it had to be discontinued (moderate doses only had been given), on account of shivering, cyanosis, and depression of pulse. It produces abundant perspirations, but not so copious as in the case of antifebrin. As a practical point, I found that in patients with enteric fever, complicated with much bronchitis, a smaller dose than usual was generally efficient in reducing temperature. It is a valuable analgesic, and may give relief when antipyrin has failed; it is, however, not so efficient as the latter in megrim.]

**Treatment of Tabes Dorsalis by Suspension.**—M. Charcot has lately made some observations on the treatment of tabes dorsalis, and of some other nervous diseases, by the method of suspension originally advocated and carried out by Dr. Motchoukowsky, of Odessa, in 1883.

The plan of treatment consists in the suspension of the patient by the help of the apparatus used in putting on a Sayre's plaster-of-Paris jacket. At first, the patient is suspended for half a minute, and afterwards for half a minute longer on each occasion until a maximum period of three or four minutes is reached. It is well to raise the patient's arms every 15 or 20 seconds, in order that more effectual traction shall be exercised on the spinal column.

Out of 14 patients thus treated, remarkable improvement took place in eight: all the cases were undoubted instances of tabes dorsalis.

An analysis of the results obtained shows that improvement in walking, when inco-ordination exists, takes place from the first, lasting at first only two or three hours; but after eight or ten suspensions, remaining constant. After about 20—30 sittings, Romberg's symptom disappears. Disorders of micturition next show improvement; and with this lightning-pains are ameliorated, decreasing in intensity and coming on less often. If there is impotence, sexual desire and power of erection return after a time. Anæsthetic areas often disappear, sleep is better, and the general state of health improves. Improvement generally advances *pari passu* with the duration of treatment. In one severe case, in which the disease came on at the age of 32, no benefit was obtained. In none of the cases did the knee-jerk return, nor did the pupillary symptoms alter.

In two cases of neurasthenia, sexual power was regained: in a case of disseminated sclerosis, the treatment appeared to do harm; for after two suspensions, spasmodic paraplegia came on and lasted for three days.

M. Charcot concludes that further experience is necessary to determine the value of this method of treatment, but that the results obtained during the three months are most encouraging in a disease for the relief of which we have hitherto been able to do so little.—*Le Progrès médical*, January 19th, 1889.

**Action of Strophanthus.**—M. G. Sée recently read before the Academy of Medicine the results of experiments made with strophanthine, an alkaloid of strophanthus. The animals experimented on were rabbits and dogs; the dose employed was 1½ milligrammes for the former, and 4 milligrammes for the latter. Soon after injection a more or less marked slowing of the heart, with rise of blood-pressure, takes place; this is followed by acceleration of the heart-beats. In the second period, which lasts until the heart ceases to beat, the cardiac action becomes irregular; short periods of acceleration are followed by more persistent periods of slowing, with abortive systolic contractions. The blood-pressure remains constantly high, but falls just before the end; sometimes a still further considerable rise of pressure occurs, with a sudden drop when the heart ceases to beat. This rise of the general blood-pressure was not affected by the changes in the force and frequency of the heart-beats; and further experiments showed that it was to be attributed to a direct action of the strophanthine circulating in the blood on the muscle-fibres of the arterial coats or on the nervous ganglia contained in them.

In man, strophanthine produced the best effects in heart-failure, in mitral regurgitation, or in simple dilatation of the heart from different causes: the pulse regained its regularity, dyspnœa ceased, and diuresis was set up. In disease of the aortic valves no good results were obtained, and in angina pectoris strophanthine was positively injurious. On the other hand, M. Sée found that the tinctures of strophanthus in common use were of little value, neither relieving dyspnœa, nor producing diuresis. Preparations from the plant, such as the tinctures, are very uncertain, and vary both in their physiological effects, and in the proportion of strophanthine—their only active principle—which they contain. He prefers, therefore, to prescribe the alkaloid, since the exact dose and the physiological action are then accurately known.

M. Dujardin-Beaumetz considered that, speaking generally, the use of the alkaloids was preferable to that of the extracts of plants; but in the particular case of cardiac remedies, the preparations of digitalis and strophanthus gave better results than digitaline and strophanthine. He had obtained excellent results from the administration of strophanthus in chronic diseases of the kidneys.

M. Constantine Paul and M. Bucquoy stated that strophanthus was much to be preferred to strophanthine. According to M. Bucquoy, strophanthus increases the force of the ventricular systole and the amount of the urinary secretion. In lesions of the mitral valve with a failing left ventricle, it always relieves symptoms, and often re-establishes compensation. It is useful in aortic lesions when the ventricle begins to flag, and gives excellent results in Graves' disease. Strophanthus is well borne, does not accumulate, its action is speedy, and it can be administered for some time without losing its effects—which, indeed, often persist for some time after it has been discontinued. The only symptom of intolerance is diarrhœa.—*Gazette des hôpitaux*, November 15, 1888; January 17, 24, and 31, 1889.

**Diuretic Action of Calomel.**—The diuretic properties of mercurial preparations were known to the ancient writers Boerhaave, Paracelsus, Franck, and Hoffman; but appear to have been completely forgotten until Jendrassik recalled attention to them in 1886. Since then there have been numerous communications on the subject. Calomel produces good effects in anasarca when this is of cardiac origin. In the dropsy of albuminuric patients it is not so efficacious. In order that calomel may produce diuresis, the kidneys must be in good condition.

The following is the best method of administration: three grains of calomel are given three times a day for three or four days; it is then best to stop the drug, whether the desired effect has been produced or not. After a week a fresh trial may be made; if this also fails, the remedy is to be given up. Some authors have continued the administration of calomel for fifteen days; but this is imprudent, and may be dangerous. Toxic accidents are more likely to occur when diuresis is not obtained. At the end of two or three days, the action of the calomel may increase the quantity of urine secreted by 300 c.cc.; for instance, to a total of 3,000 or 4,000 c.cc. in the twenty-four hours. If the diuretic action does not show itself by the fourth day, it is not likely to appear later. Purgation often accompanies diuresis; the greater the purgation, the less is the diuretic action of the drug. When they are not excessive, the intestinal evacuations may obviously be of advantage. Salivation is to be carefully avoided: by the concurrent administration of a little opium, and the use of a chlorate of potash gargle from the first, the toxic effects of calomel can in most cases be obviated.—A. Mathien, *Gazette des hôpitaux*, January 15th, 1889.

J. Michell Clarke, M.B.

